# Bis(2,9-dimethyl-1,10-phenanthroline)copper(I) penta­cyanido­nitro­soferrate(II)

**DOI:** 10.1107/S1600536813031760

**Published:** 2013-11-27

**Authors:** Julia A. Rusanova, Olesia V. Kozachuk, Valentyna V. Semenaka, Viktoriya V. Dyakonenko

**Affiliations:** a National Taras Shevchenko University, Department of Inorganic Chemistry, Volodymyrska str. 64/13, 01601 Kyiv, Ukraine; b Institute for Scintillation Materials, "Institute for Single Crystals", National Academy of Sciences of Ukraine, Lenina ave. 60, Kharkov 61001, Ukraine

## Abstract

The asymmetric unit of the title complex [Cu(C_14_H_12_N_2_)_2_]_2_[Fe(CN)_5_(NO)], consists of a [Cu(dmp)_2_]^+^ cation (dmp is 2,9-dimethyl-1,10-phenanthroline) and half an [Fe(CN)_5_(NO)]^2−^ anion. The anion is disordered across an inversion center with the Fe^II^ ion slightly offset (*ca* 0.205Å) from the inversion center in the direction of the disordered *trans*-coordinating CN/NO ligands. The anion has a distorted octa­hedral coordination geometry. The Cu^I^ ion is coordinated by two phenanthroline ligands in a distorted tetra­hedral geometry. The dihedral angle between the phenanthroline ligands is 77.16 (4) Å. In the crystal, the cations are connected to the anions by weak C—H⋯N hydrogen bonds. In addition, weak π–π stacking inter­actions are observed, with centroid–centroid distances in the range 3.512 (3)–3.859 (3) Å.

## Related literature

For background to the direct synthesis of coordination compounds, see: Kokozay & Vassilyeva (2002 ▶); Nesterova *et al.* (2008 ▶). For the direct synthesis of heterometallic Cu-containing complexes, see: Buvaylo *et al.* (2005 ▶); Nesterova *et al.* (2004 ▶, 2005 ▶); Pryma *et al.* (2003 ▶). For the application of anionic complexes in the preparation of heterometallic compounds, see: Nikitina *et al.* (2008 ▶, 2009 ▶). For the structures of related complexes, see: Blake *et al.* (1998 ▶); Chen *et al.* (2002 ▶); Morpurgo *et al.* (1984 ▶); Cuttell *et al.* (2002 ▶); King *et al.* (2005 ▶); Soria *et al.* (2002 ▶); Shevyakova *et al.* (2002 ▶); Peresyp­kina & Vostrikova (2012 ▶).
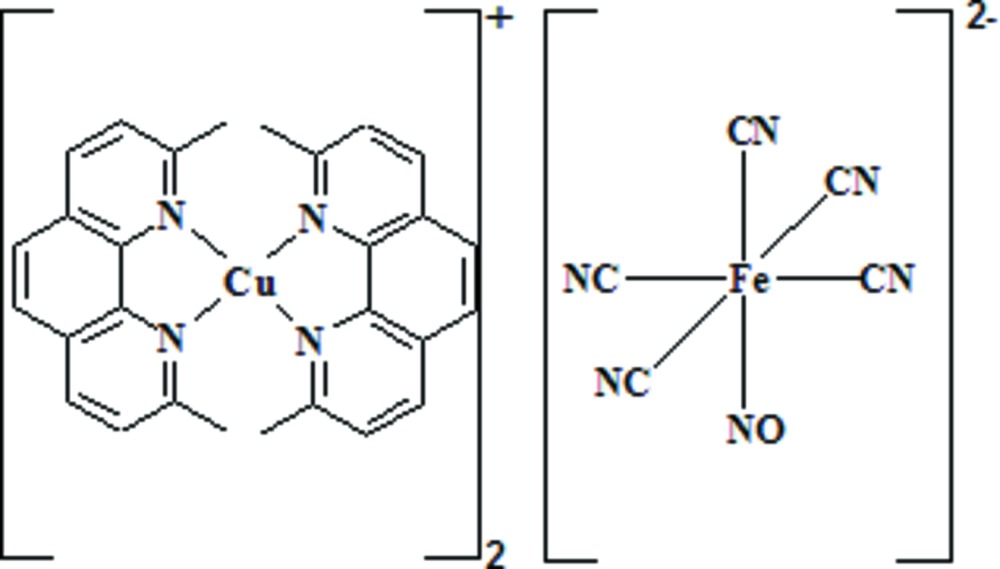


## Experimental

### 

#### Crystal data

[Cu(C_14_H_12_N_2_)_2_]_2_[Fe(CN)_5_(NO)]
*M* *_r_* = 1176.06
Triclinic, 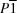

*a* = 7.371 (3) Å
*b* = 13.741 (3) Å
*c* = 15.065 (4) Å
α = 115.269 (4)°
β = 95.327 (3)°
γ = 101.323 (4)°
*V* = 1325.9 (7) Å^3^
*Z* = 1
Mo *K*α radiation
μ = 1.12 mm^−1^
*T* = 293 K
0.50 × 0.40 × 0.20 mm

#### Data collection

Oxford Diffraction Xcalibur3 diffractometer
Absorption correction: numerical (*CrysAlis PRO*; Oxford Diffraction, 2010 ▶) *T*_min_ = 0.604, *T*_max_ = 0.807
8613 measured reflections
5112 independent reflections
3100 reflections with *I* > 2σ(*I*)
*R*_int_ = 0.048

#### Refinement

*R*[*F* ^2^ > 2σ(*F* ^2^)] = 0.050
*wR*(*F* ^2^) = 0.109
*S* = 0.93
5112 reflections
377 parameters
H-atom parameters constrained
Δρ_max_ = 0.38 e Å^−3^
Δρ_min_ = −0.31 e Å^−3^

### 

Data collection: *CrysAlis PRO* (Oxford Diffraction, 2010 ▶); cell refinement: *CrysAlis PRO*; data reduction: *CrysAlis PRO*; program(s) used to solve structure: *SHELXTL* (Sheldrick, 2008 ▶); program(s) used to refine structure: *SHELXTL*; molecular graphics: *SHELXTL*; software used to prepare material for publication: *publCIF* (Westrip, 2010 ▶).

## Supplementary Material

Crystal structure: contains datablock(s) I, New_Global_Publ_Block. DOI: 10.1107/S1600536813031760/lh5667sup1.cif

Structure factors: contains datablock(s) I. DOI: 10.1107/S1600536813031760/lh5667Isup2.hkl

Additional supplementary materials:  crystallographic information; 3D view; checkCIF report

## Figures and Tables

**Table 1:** Hydrogen-bond geometry (Å, °)

*D*—H⋯*A*	*D*—H	H⋯*A*	*D*⋯*A*	*D*—H⋯*A*
C17—H17⋯N6^i^	0.93	2.55	3.393 (6)	151

Symmetry code: (i) 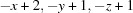.

**Fig. 1.**
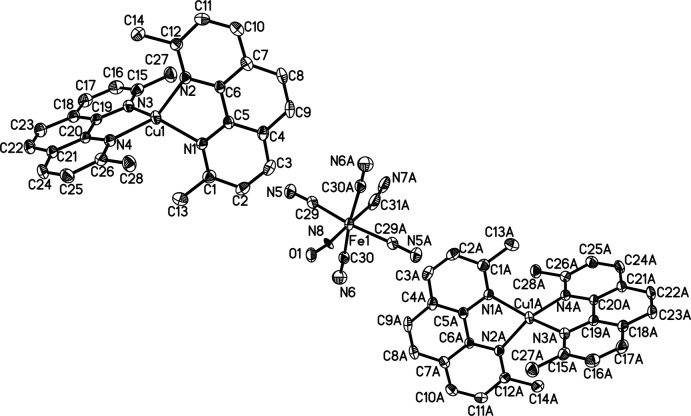
The molecular structure of the title compound with displacement ellipsoids drawn at the 30% probability level. Symmetry code (A); 2 -x,1-y,-z. The disorder is not shown.

**Fig. 2.**
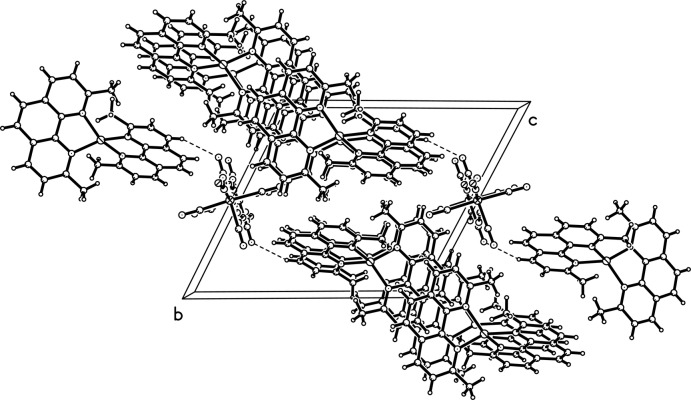
Part of the crystal structure with weak hydrogen bonds shown as dashed lines.

## supplementary crystallographic information

### 1. Comment

The title compound was obtained as part of our research in the field of direct synthesis of coordination compounds (Buvaylo *et al.*, 2005; Kokozay *et al.*, 2002; Nikitina *et al.*, 2008, 2009; Nesterova *et al.*, 2004, 2005, 2008; Pryma *et al.*,2003). Complexes of copper chelated with phenanthroline (in particular 2,9-dimethyl-1,10-phenanthroline) have attracted attention due to their longlived excited states and potential use in solar energy conversion (Blake *et al.*, 1998; Chen *et al.*, 2002; Morpurgo *et al.*, 1984; Cuttell *et al.*, 2002; King *et al.*, 2005).

In this paper we present a novel Cu/Fe heterometallic ionic complex [Cu(dmp)_2_]_2_[Fe(CN)_5_NO] which consists of discrete [Cu(dmp)_2_]^+^ and [Fe(CN)_5_NO]^2-^ ions (Fig. 1). The Cu^I^ ion adopts a distorted tetrahedral environment by coordinating with four nitrogen atoms from two dmp ligands. The dihedral angle between the two dmp ligands (77.16 (4) Å) as well as the range of Cu—N bond distances of 2.034 (3) - 2.079 (3) Å is in good agreement with the previously reported values for analagous complexes (King *et al.*, 2005 and references therein). The nitroprusside anion lies on an inversion centre and disordered over two positions so that iron atom occupies two very close positions (Fe···Fe distance is 0.410 (15) Å) corresponding to the coordination of two disordered CN and NO groups in the axial sites with very close positions. However, geometric parameters (average Fe—CN and Fe—NO bond distances of 1.96 Å and 1.63 Å respectively) are in a good agreement with literature values (Soria *et al.* (2002); Shevyakova *et al.* (2002); Peresypkina *et al.* (2012).

In the crystal, cations are connected to the anions by weak C—H···N hydrogen bonds. In addition weak π–π stacking interactions with centroid–centroid distances in the range 3.512 (3)–3.859 (3)° are observed (Fig. 2).

### 2. Experimental

Copper powder (0.04 g, 0.63 mmol), NH_4_Br (0.123 g, 1.25 mmol), Na_2_[Fe(CN)_5_(NO)]^.^2H_2_O (0.188 g, 0.63 mmol) and dmp (0.262 g, 1.26 mmol) in DMF (30 ml) were heated to 333–343 K and stirred magnetically until total dissolution of copper was observed (2.5 h). Red needle-shaped crystals suitable for X-ray crystallography was isolated from the resulting dark-red solution with addition of 2-propanol and diethyl ether in a few days. The crystals (0.1 g, yield 30%) were filtered off, washed with dry methanol, and finally dried *in vacuo* at room temperature.

### 3. Refinement

All non-hydrogen atoms were refined isotropically. All hydrogen atoms were placed at calculated position and refined in a riding-model approximation. The symmetry realted Fe atoms are offset from an inversion centre by 0.214 Å and were refined with multiplicity 0.5. Atoms of disordered CN and NO groups occupy close positions and also were refined with multiplicity 0.5.

### Crystal data

**Table:**

[Cu(C_14_H_12_N_2_)_2_]_2_[Fe(CN)_5_(NO)]	*Z* = 1
*M**_r_* = 1176.06	*F*(000) = 604
Triclinic, *P*1	*D*_x_ = 1.473 Mg m^−^^3^
Hall symbol: -P 1	Mo *K*α radiation, λ = 0.71073 Å
*a* = 7.371 (3) Å	Cell parameters from 4215 reflections
*b* = 13.741 (3) Å	θ = 2.7–24.8°
*c* = 15.065 (4) Å	µ = 1.12 mm^−^^1^
α = 115.269 (4)°	*T* = 293 K
β = 95.327 (3)°	Needle-shaped, red
γ = 101.323 (4)°	0.50 × 0.40 × 0.20 mm
*V* = 1325.9 (7) Å^3^	

### Data collection

**Table:**

Oxford Diffraction Xcalibur3 diffractometer	5112 independent reflections
Radiation source: Enhance (Mo) X-ray Source	3100 reflections with *I* > 2σ(*I*)
Graphite monochromator	*R*_int_ = 0.048
Detector resolution: 16.1827 pixels mm^-1^	θ_max_ = 26.0°, θ_min_ = 2.9°
ω–scans	*h* = −8→9
Absorption correction: numerical (*CrysAlis PRO*; Oxford Diffraction, 2010)	*k* = −16→16
*T*_min_ = 0.604, *T*_max_ = 0.807	*l* = −18→15
8613 measured reflections	

### Refinement

**Table:**

Refinement on *F*^2^	Primary atom site location: structure-invariant direct methods
Least-squares matrix: full	Secondary atom site location: difference Fourier map
*R*[*F*^2^ > 2σ(*F*^2^)] = 0.050	Hydrogen site location: inferred from neighbouring sites
*wR*(*F*^2^) = 0.109	H-atom parameters constrained
*S* = 0.93	*w* = 1/[σ^2^(*F*_o_^2^) + (0.0468*P*)^2^] where *P* = (*F*_o_^2^ + 2*F*_c_^2^)/3
5112 reflections	(Δ/σ)_max_ = 0.001
377 parameters	Δρ_max_ = 0.38 e Å^−^^3^
0 restraints	Δρ_min_ = −0.31 e Å^−^^3^

### Special details

**Table:**

Geometry. All e.s.d.'s (except the e.s.d. in the dihedral angle between two l.s. planes) are estimated using the full covariance matrix. The cell e.s.d.'s are taken into account individually in the estimation of e.s.d.'s in distances, angles and torsion angles; correlations between e.s.d.'s in cell parameters are only used when they are defined by crystal symmetry. An approximate (isotropic) treatment of cell e.s.d.'s is used for estimating e.s.d.'s involving l.s. planes.
Refinement. Refinement of *F*^2^ against ALL reflections. The weighted *R*-factor *wR* and goodness of fit *S* are based on *F*^2^, conventional *R*-factors *R* are based on *F*, with *F* set to zero for negative *F*^2^. The threshold expression of *F*^2^ > σ(*F*^2^) is used only for calculating *R*-factors(gt) *etc*. and is not relevant to the choice of reflections for refinement. *R*-factors based on *F*^2^ are statistically about twice as large as those based on *F*, and *R*- factors based on ALL data will be even larger.

### Fractional atomic coordinates and isotropic or equivalent isotropic displacement parameters (Å^2^)

**Table:**

	*x*	*y*	*z*	*U*_iso_*/*U*_eq_	Occ. (<1)
Cu1	0.24477 (7)	0.18237 (4)	0.30785 (3)	0.04709 (18)	
Fe1	0.9747 (13)	0.5030 (13)	0.0033 (11)	0.0423 (14)	0.50
N1	0.3564 (4)	0.2540 (2)	0.2232 (2)	0.0377 (7)	
N2	0.1731 (4)	0.0423 (2)	0.1741 (2)	0.0369 (7)	
N3	0.3933 (4)	0.1682 (2)	0.4241 (2)	0.0379 (7)	
N4	0.1040 (4)	0.2617 (2)	0.4159 (2)	0.0377 (7)	
C1	0.4355 (5)	0.3608 (3)	0.2474 (3)	0.0464 (10)	
C2	0.5081 (5)	0.3909 (3)	0.1771 (3)	0.0551 (11)	
H2	0.5610	0.4656	0.1954	0.066*	
C3	0.5025 (5)	0.3129 (4)	0.0833 (3)	0.0542 (11)	
H3	0.5561	0.3335	0.0383	0.065*	
C4	0.4148 (5)	0.2001 (3)	0.0542 (3)	0.0407 (9)	
C5	0.3423 (4)	0.1751 (3)	0.1269 (3)	0.0345 (8)	
C6	0.2450 (4)	0.0621 (3)	0.1008 (3)	0.0336 (8)	
C7	0.2248 (5)	−0.0217 (3)	0.0027 (3)	0.0381 (9)	
C8	0.3039 (5)	0.0072 (4)	−0.0683 (3)	0.0481 (10)	
H8	0.2923	−0.0483	−0.1331	0.058*	
C9	0.3950 (5)	0.1129 (4)	−0.0439 (3)	0.0492 (10)	
H9	0.4457	0.1292	−0.0918	0.059*	
C10	0.1271 (5)	−0.1296 (3)	−0.0187 (3)	0.0458 (10)	
H10	0.1092	−0.1876	−0.0829	0.055*	
C11	0.0583 (5)	−0.1498 (3)	0.0538 (3)	0.0460 (10)	
H11	−0.0052	−0.2220	0.0394	0.055*	
C12	0.0824 (5)	−0.0622 (3)	0.1510 (3)	0.0383 (9)	
C13	0.4435 (6)	0.4460 (3)	0.3521 (3)	0.0654 (12)	
H13A	0.5696	0.4934	0.3804	0.098*	
H13B	0.4081	0.4091	0.3919	0.098*	
H13C	0.3580	0.4902	0.3512	0.098*	
C14	0.0096 (6)	−0.0831 (3)	0.2332 (3)	0.0559 (11)	
H14A	0.0870	−0.1209	0.2545	0.084*	
H14B	−0.1182	−0.1286	0.2086	0.084*	
H14C	0.0137	−0.0133	0.2888	0.084*	
C15	0.5321 (5)	0.1207 (3)	0.4270 (3)	0.0454 (10)	
C16	0.6193 (6)	0.1264 (3)	0.5157 (3)	0.0547 (11)	
H16	0.7197	0.0947	0.5157	0.066*	
C17	0.5581 (6)	0.1786 (3)	0.6032 (3)	0.0549 (11)	
H17	0.6176	0.1830	0.6625	0.066*	
C18	0.4051 (5)	0.2251 (3)	0.6023 (3)	0.0451 (10)	
C19	0.3273 (5)	0.2190 (3)	0.5108 (3)	0.0394 (9)	
C20	0.1730 (5)	0.2687 (3)	0.5074 (3)	0.0358 (9)	
C21	0.1046 (5)	0.3217 (3)	0.5935 (3)	0.0420 (9)	
C22	0.1808 (6)	0.3230 (3)	0.6840 (3)	0.0528 (11)	
H22	0.1298	0.3551	0.7405	0.063*	
C23	0.3275 (6)	0.2779 (3)	0.6893 (3)	0.0548 (11)	
H23	0.3780	0.2813	0.7498	0.066*	
C24	−0.0412 (6)	0.3721 (3)	0.5857 (3)	0.0516 (11)	
H24	−0.0922	0.4082	0.6412	0.062*	
C25	−0.1066 (6)	0.3674 (3)	0.4963 (3)	0.0516 (10)	
H25	−0.2005	0.4022	0.4912	0.062*	
C26	−0.0338 (5)	0.3106 (3)	0.4117 (3)	0.0417 (9)	
C27	0.5909 (6)	0.0609 (4)	0.3308 (3)	0.0619 (12)	
H27A	0.5252	−0.0168	0.3005	0.093*	
H27B	0.7245	0.0690	0.3432	0.093*	
H27C	0.5611	0.0917	0.2866	0.093*	
C28	−0.1065 (6)	0.3050 (4)	0.3126 (3)	0.0544 (11)	
H28A	−0.0458	0.2601	0.2627	0.082*	
H28B	−0.0798	0.3788	0.3182	0.082*	
H28C	−0.2404	0.2723	0.2937	0.082*	
C29	1.0149 (5)	0.4533 (3)	0.1060 (3)	0.0446 (9)	
N5	1.0271 (5)	0.4286 (3)	0.1677 (3)	0.0631 (10)	
C30	1.1809 (6)	0.6397 (3)	0.0926 (3)	0.0440 (9)	
N6	1.2879 (5)	0.7213 (3)	0.1470 (3)	0.0626 (10)	
C31	0.812 (5)	0.559 (2)	0.036 (2)	0.070 (5)	0.50
N7	0.681 (4)	0.603 (2)	0.0546 (17)	0.070 (5)	0.50
N8	0.805 (3)	0.5579 (11)	0.0446 (12)	0.026 (3)	0.50
O1	0.688 (3)	0.5921 (16)	0.0752 (11)	0.055 (3)	0.50

### Atomic displacement parameters (Å^2^)

**Table:**

	*U*^11^	*U*^22^	*U*^33^	*U*^12^	*U*^13^	*U*^23^
Cu1	0.0659 (3)	0.0491 (3)	0.0278 (3)	0.0167 (2)	0.0157 (2)	0.0169 (2)
Fe1	0.057 (4)	0.0453 (14)	0.0274 (15)	0.012 (3)	0.009 (3)	0.0199 (12)
N1	0.0449 (17)	0.0362 (18)	0.0314 (17)	0.0069 (14)	0.0073 (14)	0.0166 (14)
N2	0.0400 (16)	0.0401 (18)	0.0323 (17)	0.0114 (14)	0.0067 (14)	0.0176 (14)
N3	0.0423 (16)	0.0398 (18)	0.0323 (17)	0.0120 (15)	0.0089 (15)	0.0162 (14)
N4	0.0433 (17)	0.0362 (17)	0.0302 (17)	0.0064 (14)	0.0041 (15)	0.0143 (14)
C1	0.047 (2)	0.045 (2)	0.045 (2)	0.0067 (19)	0.006 (2)	0.0210 (19)
C2	0.059 (2)	0.046 (3)	0.064 (3)	0.005 (2)	0.015 (2)	0.032 (2)
C3	0.056 (2)	0.065 (3)	0.053 (3)	0.009 (2)	0.019 (2)	0.038 (2)
C4	0.0384 (19)	0.057 (3)	0.034 (2)	0.0162 (19)	0.0086 (18)	0.0255 (19)
C5	0.0341 (18)	0.043 (2)	0.0300 (19)	0.0145 (17)	0.0068 (16)	0.0184 (17)
C6	0.0358 (18)	0.040 (2)	0.0295 (19)	0.0158 (17)	0.0084 (17)	0.0175 (16)
C7	0.0377 (19)	0.048 (2)	0.030 (2)	0.0209 (18)	0.0048 (17)	0.0153 (17)
C8	0.050 (2)	0.063 (3)	0.027 (2)	0.017 (2)	0.0100 (19)	0.0155 (19)
C9	0.046 (2)	0.080 (3)	0.031 (2)	0.022 (2)	0.0159 (19)	0.030 (2)
C10	0.047 (2)	0.048 (3)	0.036 (2)	0.020 (2)	0.0036 (19)	0.0117 (19)
C11	0.045 (2)	0.037 (2)	0.053 (3)	0.0118 (18)	0.004 (2)	0.017 (2)
C12	0.042 (2)	0.039 (2)	0.039 (2)	0.0130 (18)	0.0068 (18)	0.0210 (18)
C13	0.081 (3)	0.043 (3)	0.058 (3)	0.010 (2)	0.013 (3)	0.013 (2)
C14	0.067 (3)	0.048 (3)	0.061 (3)	0.015 (2)	0.022 (2)	0.031 (2)
C15	0.049 (2)	0.045 (2)	0.042 (2)	0.005 (2)	0.001 (2)	0.024 (2)
C16	0.054 (2)	0.055 (3)	0.058 (3)	0.017 (2)	0.003 (2)	0.029 (2)
C17	0.067 (3)	0.055 (3)	0.041 (3)	0.008 (2)	−0.005 (2)	0.027 (2)
C18	0.058 (2)	0.041 (2)	0.033 (2)	0.006 (2)	0.004 (2)	0.0179 (18)
C19	0.052 (2)	0.034 (2)	0.029 (2)	0.0044 (18)	0.0058 (18)	0.0152 (16)
C20	0.047 (2)	0.033 (2)	0.0260 (19)	0.0058 (17)	0.0075 (18)	0.0142 (16)
C21	0.053 (2)	0.039 (2)	0.031 (2)	0.0065 (19)	0.0102 (19)	0.0147 (17)
C22	0.072 (3)	0.054 (3)	0.026 (2)	0.009 (2)	0.014 (2)	0.0142 (19)
C23	0.080 (3)	0.053 (3)	0.026 (2)	0.009 (2)	0.006 (2)	0.0180 (19)
C24	0.064 (3)	0.050 (3)	0.035 (2)	0.013 (2)	0.022 (2)	0.0115 (19)
C25	0.058 (2)	0.050 (3)	0.050 (3)	0.023 (2)	0.017 (2)	0.020 (2)
C26	0.046 (2)	0.040 (2)	0.039 (2)	0.0096 (19)	0.0086 (19)	0.0193 (18)
C27	0.067 (3)	0.070 (3)	0.060 (3)	0.031 (2)	0.027 (2)	0.032 (2)
C28	0.062 (2)	0.065 (3)	0.041 (2)	0.024 (2)	0.009 (2)	0.026 (2)
C29	0.055 (2)	0.045 (2)	0.037 (2)	0.0171 (19)	0.015 (2)	0.0185 (19)
N5	0.093 (3)	0.067 (3)	0.044 (2)	0.028 (2)	0.020 (2)	0.0347 (19)
C30	0.058 (2)	0.048 (3)	0.033 (2)	0.021 (2)	0.009 (2)	0.0222 (19)
N6	0.069 (2)	0.062 (3)	0.052 (2)	0.016 (2)	−0.003 (2)	0.025 (2)
C31	0.087 (9)	0.109 (9)	0.050 (8)	0.044 (7)	0.017 (7)	0.061 (7)
N7	0.087 (9)	0.109 (9)	0.050 (8)	0.044 (7)	0.017 (7)	0.061 (7)
N8	0.039 (5)	0.022 (4)	0.011 (5)	0.012 (4)	0.009 (4)	−0.001 (4)
O1	0.082 (6)	0.087 (7)	0.027 (5)	0.057 (5)	0.026 (5)	0.036 (5)

### Geometric parameters (Å, º)

**Table:**

Cu1—N2	2.034 (3)	C13—H13A	0.9600
Cu1—N4	2.039 (3)	C13—H13B	0.9600
Cu1—N1	2.053 (3)	C13—H13C	0.9600
Cu1—N3	2.079 (3)	C14—H14A	0.9600
Fe1—Fe1^i^	0.410 (15)	C14—H14B	0.9600
Fe1—C31	1.56 (4)	C14—H14C	0.9600
Fe1—N8	1.625 (19)	C15—C16	1.392 (5)
Fe1—C30^i^	1.908 (15)	C15—C27	1.484 (5)
Fe1—C31^i^	1.96 (4)	C16—C17	1.373 (6)
Fe1—C29	1.961 (16)	C16—H16	0.9300
Fe1—C29^i^	1.981 (16)	C17—C18	1.403 (5)
Fe1—C30	1.998 (15)	C17—H17	0.9300
Fe1—N8^i^	2.03 (2)	C18—C19	1.404 (5)
N1—C1	1.341 (4)	C18—C23	1.435 (5)
N1—C5	1.370 (4)	C19—C20	1.446 (5)
N2—C12	1.334 (4)	C20—C21	1.388 (5)
N2—C6	1.372 (4)	C21—C24	1.410 (5)
N3—C15	1.324 (5)	C21—C22	1.416 (5)
N3—C19	1.379 (4)	C22—C23	1.360 (6)
N4—C26	1.334 (4)	C22—H22	0.9300
N4—C20	1.380 (4)	C23—H23	0.9300
C1—C2	1.404 (5)	C24—C25	1.358 (5)
C1—C13	1.497 (5)	C24—H24	0.9300
C2—C3	1.351 (5)	C25—C26	1.401 (5)
C2—H2	0.9300	C25—H25	0.9300
C3—C4	1.409 (5)	C26—C28	1.502 (5)
C3—H3	0.9300	C27—H27A	0.9600
C4—C5	1.399 (5)	C27—H27B	0.9600
C4—C9	1.421 (5)	C27—H27C	0.9600
C5—C6	1.439 (5)	C28—H28A	0.9600
C6—C7	1.403 (5)	C28—H28B	0.9600
C7—C10	1.397 (5)	C28—H28C	0.9600
C7—C8	1.425 (5)	C29—N5	1.120 (4)
C8—C9	1.346 (5)	C29—Fe1^i^	1.981 (16)
C8—H8	0.9300	C30—N6	1.140 (5)
C9—H9	0.9300	C30—Fe1^i^	1.908 (15)
C10—C11	1.354 (5)	C31—N7	1.23 (5)
C10—H10	0.9300	C31—Fe1^i^	1.96 (4)
C11—C12	1.410 (5)	N8—O1	1.11 (3)
C11—H11	0.9300	N8—Fe1^i^	2.03 (2)
C12—C14	1.507 (5)		
			
N2—Cu1—N4	135.31 (11)	C8—C9—H9	119.6
N2—Cu1—N1	82.52 (12)	C4—C9—H9	119.6
N4—Cu1—N1	121.22 (12)	C11—C10—C7	120.1 (4)
N2—Cu1—N3	114.85 (12)	C11—C10—H10	120.0
N4—Cu1—N3	82.49 (12)	C7—C10—H10	120.0
N1—Cu1—N3	126.67 (11)	C10—C11—C12	120.4 (4)
Fe1^i^—Fe1—C31	165 (5)	C10—C11—H11	119.8
Fe1^i^—Fe1—N8	166 (5)	C12—C11—H11	119.8
C31—Fe1—N8	4.6 (19)	N2—C12—C11	121.3 (3)
Fe1^i^—Fe1—C30^i^	97 (4)	N2—C12—C14	117.4 (3)
C31—Fe1—C30^i^	96.8 (11)	C11—C12—C14	121.3 (3)
N8—Fe1—C30^i^	96.8 (7)	C1—C13—H13A	109.5
Fe1^i^—Fe1—C31^i^	12 (4)	C1—C13—H13B	109.5
C31—Fe1—C31^i^	176.8 (10)	H13A—C13—H13B	109.5
N8—Fe1—C31^i^	175.8 (18)	C1—C13—H13C	109.5
C30^i^—Fe1—C31^i^	86.0 (10)	H13A—C13—H13C	109.5
Fe1^i^—Fe1—C29	87 (4)	H13B—C13—H13C	109.5
C31—Fe1—C29	99.8 (13)	C12—C14—H14A	109.5
N8—Fe1—C29	95.2 (10)	C12—C14—H14B	109.5
C30^i^—Fe1—C29	93.0 (7)	H14A—C14—H14B	109.5
C31^i^—Fe1—C29	81.6 (11)	C12—C14—H14C	109.5
Fe1^i^—Fe1—C29^i^	81 (4)	H14A—C14—H14C	109.5
C31—Fe1—C29^i^	91.8 (14)	H14B—C14—H14C	109.5
N8—Fe1—C29^i^	96.4 (10)	N3—C15—C16	121.8 (4)
C30^i^—Fe1—C29^i^	88.4 (6)	N3—C15—C27	116.8 (3)
C31^i^—Fe1—C29^i^	86.7 (11)	C16—C15—C27	121.3 (4)
C29—Fe1—C29^i^	168.1 (5)	C17—C16—C15	120.5 (4)
Fe1^i^—Fe1—C30	72 (4)	C17—C16—H16	119.8
C31—Fe1—C30	94.9 (12)	C15—C16—H16	119.8
N8—Fe1—C30	94.9 (9)	C16—C17—C18	119.5 (4)
C30^i^—Fe1—C30	168.2 (5)	C16—C17—H17	120.3
C31^i^—Fe1—C30	82.3 (9)	C18—C17—H17	120.3
C29—Fe1—C30	86.5 (6)	C17—C18—C19	117.0 (3)
C29^i^—Fe1—C30	89.7 (6)	C17—C18—C23	123.4 (4)
Fe1^i^—Fe1—N8^i^	11 (4)	C19—C18—C23	119.6 (4)
C31—Fe1—N8^i^	174.2 (19)	N3—C19—C18	122.7 (3)
N8—Fe1—N8^i^	177.2 (10)	N3—C19—C20	118.5 (3)
C30^i^—Fe1—N8^i^	85.9 (6)	C18—C19—C20	118.8 (3)
C31^i^—Fe1—N8^i^	3.6 (15)	N4—C20—C21	123.7 (3)
C29—Fe1—N8^i^	85.2 (8)	N4—C20—C19	116.3 (3)
C29^i^—Fe1—N8^i^	83.1 (7)	C21—C20—C19	120.1 (3)
C30—Fe1—N8^i^	82.4 (5)	C20—C21—C24	117.1 (4)
C1—N1—C5	118.1 (3)	C20—C21—C22	120.0 (4)
C1—N1—Cu1	130.8 (2)	C24—C21—C22	122.9 (4)
C5—N1—Cu1	111.1 (2)	C23—C22—C21	120.9 (4)
C12—N2—C6	118.1 (3)	C23—C22—H22	119.6
C12—N2—Cu1	130.3 (2)	C21—C22—H22	119.6
C6—N2—Cu1	111.5 (2)	C22—C23—C18	120.6 (4)
C15—N3—C19	118.5 (3)	C22—C23—H23	119.7
C15—N3—Cu1	131.4 (3)	C18—C23—H23	119.7
C19—N3—Cu1	110.1 (2)	C25—C24—C21	119.4 (4)
C26—N4—C20	117.4 (3)	C25—C24—H24	120.3
C26—N4—Cu1	130.0 (2)	C21—C24—H24	120.3
C20—N4—Cu1	112.6 (2)	C24—C25—C26	120.5 (4)
N1—C1—C2	121.0 (4)	C24—C25—H25	119.7
N1—C1—C13	117.3 (3)	C26—C25—H25	119.7
C2—C1—C13	121.7 (4)	N4—C26—C25	121.9 (4)
C3—C2—C1	121.1 (4)	N4—C26—C28	117.5 (3)
C3—C2—H2	119.5	C25—C26—C28	120.6 (4)
C1—C2—H2	119.5	C15—C27—H27A	109.5
C2—C3—C4	119.4 (4)	C15—C27—H27B	109.5
C2—C3—H3	120.3	H27A—C27—H27B	109.5
C4—C3—H3	120.3	C15—C27—H27C	109.5
C5—C4—C3	117.0 (3)	H27A—C27—H27C	109.5
C5—C4—C9	119.5 (4)	H27B—C27—H27C	109.5
C3—C4—C9	123.5 (4)	C26—C28—H28A	109.5
N1—C5—C4	123.2 (3)	C26—C28—H28B	109.5
N1—C5—C6	117.1 (3)	H28A—C28—H28B	109.5
C4—C5—C6	119.7 (3)	C26—C28—H28C	109.5
N2—C6—C7	123.2 (3)	H28A—C28—H28C	109.5
N2—C6—C5	117.4 (3)	H28B—C28—H28C	109.5
C7—C6—C5	119.4 (3)	N5—C29—Fe1	174.5 (4)
C10—C7—C6	116.9 (3)	N5—C29—Fe1^i^	173.2 (5)
C10—C7—C8	124.0 (3)	N6—C30—Fe1^i^	173.6 (4)
C6—C7—C8	119.0 (3)	N6—C30—Fe1	174.6 (4)
C9—C8—C7	121.6 (4)	N7—C31—Fe1	175 (3)
C9—C8—H8	119.2	N7—C31—Fe1^i^	173 (3)
C7—C8—H8	119.2	O1—N8—Fe1	176.5 (19)
C8—C9—C4	120.8 (4)	O1—N8—Fe1^i^	176.7 (16)

Symmetry code: (i) −*x*+2, −*y*+1, −*z*.

### Hydrogen-bond geometry (Å, º)

**Table:**

*D*—H···*A*	*D*—H	H···*A*	*D*···*A*	*D*—H···*A*
C17—H17···N6^ii^	0.93	2.55	3.393 (6)	151

Symmetry code: (ii) −*x*+2, −*y*+1, −*z*+1.
